# Predicting cognitive decline in cognitively impaired patients with ischemic stroke with high risk of cerebral hemorrhage: a machine learning approach

**DOI:** 10.3389/fneur.2025.1569073

**Published:** 2025-07-25

**Authors:** Eun Namgung, Young Sun Kim, Sun U. Kwon, Dong-Wha Kang

**Affiliations:** ^1^Asan Institute for Life Sciences, Asan Medical Center, Seoul, Republic of Korea; ^2^Nunaps Inc., Seoul, Republic of Korea; ^3^Department of Neurology, Asan Medical Center, University of Ulsan College of Medicine, Seoul, Republic of Korea

**Keywords:** machine learning, cognitive decline, ischemic stroke, cerebral hemorrhage, post-stroke cognitive impairment

## Abstract

**Background and objective:**

Cognitive decline progresses rapidly in stroke patients, increasing risks of stroke recurrence. Predicting deterioration within a year in patients with poststroke cognitive impairment (PSCI) could guide targeted interventions for dementia prevention and better prognosis. In this PreventIon of CArdiovascular events in iSchemic Stroke patients with high risk of cerebral hemOrrhage for reducing cognitive decline substudy, machine learning on clinical and imaging data was used to predict cognitive decline over 9 months in PSCI patients.

**Methods:**

This retrospective study included 109 patients with acute ischemic stroke and high-risk cerebral hemorrhage with PSCI (baseline Korean-Mini Mental Status Examination [K-MMSE] < 24), along with baseline clinical imaging and K-MMSE assessments at baseline and after 9 months. Four machine learning algorithms were trained, Categorical Boosting (CatBoost), Adaptive Boosting (AdaBoost), eXtreme Gradient Boosting (XGBoost), and logistic regression, to predict cognitive decliners, defined as a decline of ≥3 K-MMSE points over 9 months, and ranked variable importance using the SHapley Additive exPlanations methodology.

**Results:**

CatBoost outperformed the other models in classifying cognitive decliners within 9 months. In the test set, CatBoost achieved a mean area under the curve (AUC) of 0.897, with an accuracy of 0.873; other models performed as follows: logistic regression (AUC 0.775), AdaBoost (AUC 0.767), and XGBoost (AUC 0.722). Higher baseline K-MMSE scores (total, language, orientation to place, and recall), longer interval between stroke and baseline K-MMSE, initial National Institutes of Health Stroke Scale scores, and lesion volume ratio were identified as key predictors of cognitive decline in CatBoost. Cognitive decliners showed longer interval between stroke onset and pharmacotherapy initiation than non-decliners.

**Conclusion:**

CatBoost effectively recognized patients with ischemic stroke at high risk of cognitive decline over 9 months. Recognizing these high-risk individuals and their risk and protective factors allows for timely and targeted interventions to improve prognosis in PSCI patients.

## 1 Introduction

Stroke, which causes long-term disability, is a global health challenge (1). Cognitive impairment frequently follows a stroke, remarkably exacerbating disability and quality of life (2, 3). Individuals who have experienced a stroke typically show rapider decline in cognitive functions, increased risk of recurrent strokes, and higher mortality rates (4, 5). Recurrent strokes are considered strong predictors of cognitive decline, with affected patients exhibiting higher rates of dementia than those with a first-ever stroke (6, 7).

Higher risk of poststroke cognitive impairment (PSCI) is related to ischemic stroke with intracerebral hemorrhage or small vessel occlusive disease, requiring optimal secondary prevention (8, 9). Although conventional antiplatelet or statin therapy may increase hemorrhage risks (10, 11), cilostazol (a phosphodiesterase-3 inhibitor) or probucol (a non-statin lipid-lowering agent) may reduce the incidence of cardiovascular events without increasing hemorrhage risk (12–14). Preserving cognitive function and preventing dementia are vital for patients with ischemic stroke and high risk of cerebral hemorrhage, particularly in Asian populations due to regional stroke differences (15, 16).

Recent meta-analyses highlight the multifactorial nature of PSCI, involving vascular, demographic, and neuroanatomical factors (17). PSCI typically manifests within 3–6 months poststroke, with recovery often plateauing beyond this period (18, 19). Given the heterogeneity in cognitive trajectories, accurate prediction of cognitive deterioration beyond this window could support the development of personalized interventions aimed at preventing dementia and enhancing quality of life (17, 20). In this context, machine learning algorithms offer a promising approach by capturing non-linear interactions among baseline variables—such as stroke severity, white matter hyperintensity, and initial cognitive status—and identifying key predictors through feature importance analysis (20, 21).

In this substudy of PreventIon of CArdiovascular events in iSchemic Stroke patients with high risk of cerebral hemOrrhage for reducing COGnitive decline (PICASSO-COG) (12, 22), we propose machine learning algorithms leveraging clinical and imaging data to predict cognitive decline over a 9-month period in patients with acute ischemic stroke with cognitive impairment and high risk of cerebral hemorrhage – a population that requires timely prevention and intervention strategies.

## 2 Materials and methods

### 2.1 Participants and study design

This retrospective analysis focused on a subset of PICASSO-COG substudy, which evaluated the effects of cilostazol and/or probucol on cognitive functions in patients with ischemic stroke and high risk of cerebral hemorrhage from the PICASSO cohort (22). PICASSO (PreventIon of CArdiovascular events in iSchemic Stroke patients with high risk of cerebral hemOrrhage) trial is a multicenter, randomized, double-blind, placebo-controlled 2 × 2 factorial trial that compared the efficacy and safety of cilostazol versus aspirin, with and without probucol, for preventing hemorrhagic stroke and major vascular events ischemic in these patients (ClinicalTrials.gov, no. NCT01013532) (12). PICASSO study was approved by the site ethics committees and conducted according to Good Clinical Practice and the Declaration of Helsinki, with written consent obtained from all participants.

Key inclusion criteria for the PICASSO cohort were (1) age >20 years; (2) non-cardioembolic ischemic stroke or transient ischemic attack within the 180 days prior to screening; (3) previous intracerebral hemorrhage or multiple cerebral microbleeds based on clinical or radiological findings; and (4) asymptomatic intracerebral hemorrhage identified as a slit-like curvilinear lesion on magnetic resonance imaging, with no obvious history of intracerebral hemorrhage. Key exclusion criteria included cerebral hemorrhage within the past 6 months, contraindications to long-term antiplatelet therapy, severe cardiomyopathy or heart failure, and recent myocardial infarction or coronary procedures within the previous 4 weeks (12).

**Figure 1:**
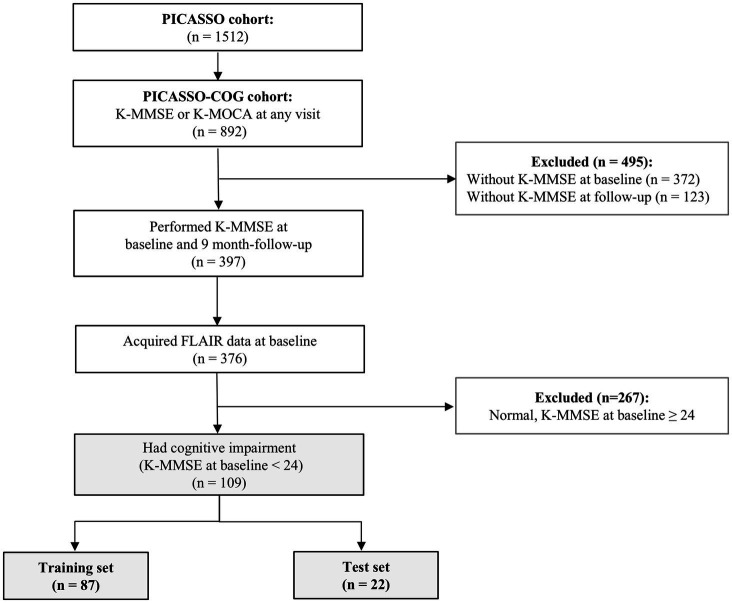
Flowchart of the inclusion criteria. The flowchart illustrates the inclusion criteria of the study participants. The participants included 109 patients who had poststroke cognitive impairment (PSCI) (K-MMSE at baseline <24). They were divided into training (87 patients) and test (22 patients) sets at an 8:2 ratio. From the patients with ischemic stroke with a history of intracerebral hemorrhage or two or more microbleeds (PICASSO cohort), patients who underwent K-MMSE at baseline between 3 and 7 months after stroke onset and 9-month follow-up and acquired baseline FLAIR data were included. PICASSO, PreventIon of CArdiovascular events in iSchemic Stroke patients with high risk of cerebral hemOrrhage; PICASSO-COG, PICASSO for reducing COGnitive decline; K-MMSE, Korean-Mini Mental Status Examination; K-MoCA, Korean-Montreal cognitive Assessment; FLAIR, fluid-attenuated inversion recovery.

Figure 1 shows the subject flow diagram. From the PICASSO cohort, 892 patients were included in the PICASSO-COG cohort after excluding those unable to undergo cognitive testing due to severe dysphasia or neurological deficits. Of these, 376 patients both the baseline evaluation (3–7 months post-stroke) and the first follow-up evaluation (≥9 months post-stroke) using the Korean Mini-Mental Status Examination (K-MMSE). Baseline Fluid-Attenuated Inversion Recovery (FLAIR) MRI scans for quantifying stroke lesion volume ratios were available for 376 of these patients. The final analysis included 109 patients with PSCI, defined by baseline K-MMSE scores <24 (23). No demographic, clinical, or imaging data were missing in the final analytic sample. Patients with incomplete cognitive assessments or missing imaging data were excluded during cohort selection; thus, imputation was not required.

### 2.2 Demographic and clinical assessment

We evaluated baseline characteristics of the participants, including demographics and clinical data (Table 1). Demographics included age, gender, and years of education, and clinical data included vital signs, lipid levels, blood glucose levels, and smoking history. We also included the concomitant pharmacotherapy assigned in the PICASSO trial (cilostazol vs. aspirin with probucol vs. no probucol) and the time between stroke onset and randomization in the PICASSO trial. Stroke-related factors included time since stroke onset, classification of ischemic events, and a high-risk index for intracerebral hemorrhage, which encompassed a history or radiological evidence of intracerebral hemorrhage and multiple microbleeds. Stroke severity was assessed using the National Institutes of Health Stroke Scale (NIHSS) score at admission (24). Fazekas scores, which indicate the extent of white matter hyperintensities (25), and lesion volume ratio, assessed by a neuroradiologist based on FLAIR images, were included as stroke-related imaging features. Volume of ischemic stroke lesions on baseline FLAIR images was automatically quantified using the lesion prediction algorithm, implemented in the Lesion Segmentation Tool (LST) within SPM12 (26). The algorithm generated a lesion probability map in which each voxel was assigned a probability of being a lesion. Voxels with a probability ≥0.5 were considered lesional. The total lesion volume (cm^3^) was calculated by multiplying the number of suprathreshold voxels by the spatial resolution of the scan. To normalize for interindividual differences in brain size, the lesion volume ratio was computed by dividing the segmented lesion volume by the total intracranial volume.

**Table 1:** Baseline characteristics between cognitive decliners and cognitive non-decliners.

Baseline characteristics	Decliners	Non-decliners	*p*
	(*n* = 27)	(*n* = 82)	
Age (years)	69.2 ± 7.9	72.3 ± 8.7	0.070
Female	15 (55.6)	52 (63.4)	0.500
Education years	6.4 ± 5.2	4.3 ± 4.2	0.080
Follow-up duration (months)	9.5 ± 1.3	9.2 ± 0.9	0.293
Months between stroke onset and baseline K-MMSE	4.9 ± 0.8	4.6 ± 0.6	0.089
K-MMSE score at baseline	23.0 ± 4.1	17.9 ± 3.8	<0.001*
Orientation to time subscore	4.0 ± 1.3	3.0 ± 1.6	0.004*
Orientation to place subscore	4.7 ± 0.6	4.1 ± 1.0	0.008*
Registration subscore	2.9 ± 0.4	2.7 ± 0.6	0.011*
Attention and calculation subscore	2.1 ± 1.8	0.9 ± 1.2	0.001*
Recall subscore	1.9 ± 1.1	1.3 ± 1.1	0.018*
Language subscore	6.9 ± 1.6	5.6 ± 1.4	<0.001*
Visuospatial ability subscore	0.5 ± 0.5	0.2 ± 0.4	0.006*
NIHSS score on admission	2.1 ± 1.9	2.1 ± 2.0	0.914
Lesion ratio in the whole brain (%)	2.9 ± 1.8	3.3 ± 1.8	0.326
Fazekas score			
0	1 (3.7)	0 (0)	0.463
1	3 (11.1)	8 (9.80)	
2	10 (37.0)	34 (41.5)	
3	13 (48.2)	40 (48.8)	
Ischemic events			
Ischemic stroke	27 (100)	80 (97.6)	<0.999
Transient ischemic event	0 (0)	2 (2.4)	
High-risk index intracerebral hemorrhage			
History of intracerebral hemorrhage	5 (18.5)	14 (17.1)	0.821
Radiological findings of intracerebral hemorrhage	5 (18.5)	21 (25.6)	
Multiple microbleeds	17 (63.0)	47 (57.3)	
Months between stroke onset and pharmacotherapy	1.4 ± 1.5	0.8 ± 1.0	0.033*
Cilostazol or aspirin therapy			
Cilostazol (100 mg/day)	16 (59.3)	46 (56.1)	0.826
Aspirin (100 mg/day)	11 (40.7)	36 (43.9)	
Addition of probucol or none			
Addition of probucol (250 mg/day)	15 (55.6)	43 (52.4)	0.827
No probucol	12 (44.4)	39 (47.6)	
Current smoking status			
Never smoked	18 (66.7)	56 (68.3)	0.978
Currently smoking	3 (11.1)	10 (12.2)	
Quit smoking in the past 3 years	2 (7.4)	6 (7.3)	
Have quit smoking for >3 years	4 (14.8)	10 (12.2)	
Systolic blood pressure (mm Hg)	136.0 ± 20.3	132.5 ± 16.7	0.491
Diastolic blood pressure (mm Hg)	80.2 ± 12.3	77.0 ± 11.0	0.238
Heart rate (beats per min)	81.8 ± 15.4	82.9 ± 13.6	0.741
Total cholesterol (mg/dL)	162.4 ± 30.6	175.1 ± 42.0	0.226
LDL cholesterol (mg/dL)	95.5 ± 29.7	110.7 ± 36.1	0.055
HDL cholesterol (mg/dL)	45.3 ± 12.7	48.0 ± 10.0	0.076
Glucose (mg/dL)	116.0 ± 49.0	116.8 ± 42.8	0.888
Hemoglobin A1c (%)	6.1 ± 1.2	6.1 ± 0.9	0.623

Baseline characteristics are indicated in mean ± standard deviation for continuous variables and number (percentage, %) for categorical variables. Continuous variables were compared using the Mann–Whitney *U* test, and categorical variables were compared using Fisher’s exact tests between decliners (*n* = 27) and non-decliners (*n* = 82).

The * symbol indicates statistical significance at *p* < 0.05.

HDL, high-density lipoprotein K-MMSE, Korean-Mini Mental Status Examination; LDL, low-density lipoprotein; NIHSS, National Institutes of Health Stroke Scale.

### 2.3 Cognitive impairment assessment

Our study focused on patients with acute ischemic stroke, cognitive impairment, and a high risk of cerebral hemorrhage— a population in urgent need of timely prevention and intervention strategies. The K-MMSE, administered by a certified neurologist, was used at baseline and follow-up to assess cognitive impairment and its progression, with total scores ranging from 0 to 30, where lower scores indicate greater impairment. Scores <24 indicated cognitive impairment (23). We focused on patients with acute ischemic stroke, cognitive impairment, and a high risk of cerebral hemorrhage— a population that requires timely prevention and intervention strategies. The K-MMSE evaluated the following seven domains: orientation to time, orientation to place, registration, attention and calculation, memory recall, language, and visuospatial ability (23). The total scores and seven domain subscores of the K-MMSE at baseline are presented in Table 1.

Baseline K-MMSE was conducted 3–7 months after stroke onset (mean ± standard deviation, 4.7 ± 0.6 months; minimum–maximum, 3.5–6.6). The follow-up K-MMSE, conducted after 9 months (9.3 ± 1.0 months; minimum–maximum, 8.3–13.3), was between 12 and 20 months after stroke onset (14.0 ± 1.2 months; minimum–maximum 12.2–19.3). A ≥ 3-point decline in K-MMSE total scores over 9 months indicated cognitive decline, according to studies suggesting significant MMSE changes of four points over 5 years and reliable annual changes of 1.3–2.7 points (27, 28).

### 2.4 Training and testing

Participants were randomly divided into a training (*n* = 87) set and a test (*n* = 22) set in an 8:2 ratio, with no significant differences observed in baseline characteristics (Table 2). Patients were categorized into two groups for labeling: those with a decrease of ≥3 K-MMSE points over 9 months received a positive label (cognitive decliners, *n* = 27, 24.7%), whereas those with *a* < 3-point decrease in K-MMSE received a negative label (cognitive non-decliners, *n* = 81, 74.3%).

**Table 2:** Baseline characteristics of training and test sets.

Baseline characteristics	Training set	Test set	*p*
	(*n* = 87)	(*n* = 22)	
Age (years)	71.4 ± 8.6	72.3 ± 8.8	0.470
Female	54 (62.1)	13 (59.1)	0.810
Education years	4.9 ± 4.6	4.7 ± 4.2	0.694
Months between stroke onset and baseline	4.7 ± 0.7	4.6 ± 0.5	0.895
K-MMSE score at baseline	19.1 ± 4.5	19.2 ± 4.4	0.922
NIHSS score on admission	2.2 ± 2.0	1.6 ± 1.6	0.190
Lesion ratio in the whole brain (%)	3.2 ± 1.8	3.5 ± 1.9	0.464
Ischemic events			
Ischemic stroke	86 (98.9)	21 (95.5)	0.364
Transient ischemic event	1 (1.2)	1 (4.6)	
High-risk index intracerebral hemorrhage			
History of intracerebral hemorrhage	16 (18.4)	3 (13.6)	0.637
Radiological findings of intracerebral hemorrhage	19 (21.8)	7 (31.8)	
Multiple microbleeds	52 (59.8)	12 (54.6)	
Months between stroke onset and pharmacotherapy	1.0 ± 1.2	0.9 ± 1.1	0.991
Cilostazol or aspirin therapy			
Cilostazol (100 mg/day)	50 (57.5)	12 (54.6)	0.814
Aspirin (100 mg/day)	37 (42.5)	10 (45.5)	
Addition of probucol or none			
Probucol (250 mg/day)	46 (52.9)	12 (54.6)	<0.999
No probucol	41 (47.1)	10 (45.5)	

Baseline characteristics are indicated in mean ± standard deviation for continuous variables and number (percentage, %) for categorical variables. Continuous variables were compared using the Mann–Whitney *U* test, and categorical variables were compared using Fisher’s exact tests between training (*n* = 87) and test (*n* = 22) sets.

K-MMSE, Korean-Mini Mental Status Examination; NIHSS, National Institutes of Health Stroke Scale.

To address class imbalance and prevent synthetic data biasing evaluation metrics, Synthetic Minority Over-sampling Technique with Tomek Links (SMOTETomek) was applied exclusively to the training set (*n* = 87) using a sampling strategy of 0.9. This method combines oversampling of the minority class (SMOTE) with undersampling of the majority class (Tomek Links) to improve class distribution while reducing noise (29). The test set (*n* = 22) preserved the original class distribution, ensuring a fair and unbiased performance assessment under real-world conditions. This separation allowed the model to learn from balanced data while maintaining external validity.

We selected the four machine learning algorithms commonly used in PSCI prediction, as identified in recent systematic reviews (20). The models trained for binary classification of cognitive decline included Categorical Boosting (CatBoost) (30), Adaptive Boosting (AdaBoost) (31), eXtreme Gradient Boosting (XGBoost) (32), and logistic regression (33). Boosting algorithms were chosen for their ability to aggregate weak learners and reduce overfitting, particularly in imbalanced datasets. Logistic regression was included for its simplicity and high interpretability.

A fivefold cross-validation scheme was implemented to evaluate model performance and optimize hyperparameters within the training set. In each fold, models were trained on four randomly selected subsets and validated on the remaining subset, known as the test set. GridSearchCV was used for AdaBoost and XGBoost, as their hyperparameter spaces are relatively small and consist of discrete values. In contrast, RandomizedSearchCV with 10 iterations was applied to CatBoost and logistic regression, which have broader or continuous hyperparameter spaces, to improve computational efficiency. StratifiedKFold was used to maintain class balance across folds, and all models were optimized based on the area under the curve (AUC). The optimal model was selected according to the average performance metrics obtained during cross-validation.

Classification performance for predicting cognitive decline at 9 months was assessed using multiple evaluation metrics, including AUC from the receiver operating characteristic (ROC) curve, accuracy, sensitivity, and specificity. Model evaluation was conducted separately on training set (*n* = 87) and independent test (*n* = 22) set. Optimal classification thresholds were determined using the Youden index (sensitivity + specificity − 1) to balance true positive and true negative rates.

All analyses were performed using Python 3.9. Key libraries included scikit-learn (v1.1.3) for training and evaluating models (logistic regression, AdaBoost); CatBoost (v1.1.1) and XGBoost (v1.4.2) for gradient boosting; and imbalanced-learn (v0.10.1) for resampling. A summary of the machine learning workflow and full package versions is provided in Supplementary Table 1.

### 2.5 Feature importance analysis

**Figure 2:**
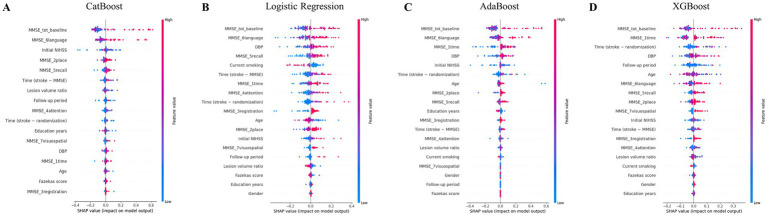
SHAP summary plot of the four machine learning models in predicting cognitive decline. The SHAP technique was used to interpret the contributing factors for the classification performance of **(A)** CatBoost, **(B)** logistic regression, **(C)** AdaBoost, and **(D)** XGBoost. Red dots in the upper right represent higher eigenvalues, which strongly contribute to predicting PSCI at 9 months, whereas blue dots in the upper left represent lower eigenvalues, also influencing PSCI prediction. AdaBoost, Adaptive Boosting; CatBoost, Categorical Boosting; DBP, diastolic blood pressure; MMSE, Korean-Mini Mental Status Examination; PSCI, poststroke cognitive impairment; SHAP, SHapley Additive exPlanations; XGBoost, Extreme Gradient Boosting.

Feature importance was analyzed using SHapley Additive exPlanations (SHAP, v0.44.1) to interpret model predictions, identify key predictors of cognitive decline, and enhance overall model transparency (34). SHAP values were used to rank input variables according to their contribution to model output. Features that consistently exhibited low SHAP values across cross-validation folds were excluded to reduce overfitting and improve interpretability. Exclusion thresholds were determined based on both cross-validated model performance and the stability of feature rankings. The final set of input features used for each model is illustrated in the SHAP summary plots (Figure 2).

### 2.6 Statistical analysis

Data are expressed as mean ± standard deviation (SD) for continuous variables and number of subjects (%, percentage) for categorical variables. The demographic and clinical characteristics were compared between training (*n* = 87) and test (*n* = 22) sets as well as between cognitive decliners (*n* = 27) and non-decliners (*n* = 82) using Mann–Whitney *U* tests for continuous variables and Fisher’s exact tests for categorical variables. These analyses were conducted to identify baseline differences and confirm that model training and evaluation sets were balanced.

## 3 Results

### 3.1 Differential baseline characteristics between cognitive decliners and non-decliners

Table 1 presents baseline characteristics of cognitive decliners and non-decliners. Cognitive decliners had a mean age of 69.2 years, 6.4 years of completed education, were 55.6% male, and had a K-MMSE total score of 23.0, indicating very mild cognitive impairment. Cognitive non-decliners had a mean age of 72.3 years, 4.2 years of completed education, were 63.4% female, and had a K-MMSE total score of 17.9, indicating moderate cognitive impairment.

Regarding baseline characteristics, cognitive decliners had a longer duration between stroke onset and randomization into pharmacotherapy of the PICASSO trial (*p* = 0.033) than non-decliners. Although this difference did not reach statistical significance, cognitive decliners exhibited trends toward younger age (*p* = 0.070), more years of education (*p* = 0.080), longer interval between stroke onset and baseline MMSE (*p* = 0.089), and lower levels of low-density lipoprotein (LDL) (*p* = 0.055) and high-density lipoprotein (HDL) (*p* = 0.076) than non-decliners (Table 1).

Notably, the baseline K-MMSE total score was significantly higher in cognitive decliners compared to non-decliners (*p* < 0.001). All baseline K-MMSE subscores were also higher in the decliner group, including orientation to time (*p* = 0.004), orientation to place (*p* = 0.008), registration (*p* = 0.011), attention and calculation (*p* = 0.001), recall (*p* = 0.018), language (*p* < 0.001), and visuospatial ability (*p* = 0.006), compared to the non-decliner group.

### 3.2 Classification of cognitive decliners in the training set

Table 3 summarizes the classification performance of the four machine learning models on cognitive decliners in the training set (*n* = 87). In the training set, a fivefold cross-validation of CatBoost yielded most superior performance than the other three machine learning models in terms of accuracy, AUC, and sensitivity. The CatBoost algorithm achieved a mean AUC of 0.966, with an accuracy of 0.897, a sensitivity of 0.909, and a specificity of 0.888.

**Table 3:** Performance of the four machine learning models in predicting cognitive decline.

Dataset	Model	AUC	Accuracy	Sensitivity	Specificity
Training set (*n* = 87)	CatBoost	0.966	0.897	0.909	0.888
	AdaBoost	0.924	0.838	0.855	0.823
	XGBoost	0.950	0.880	0.836	0.918
	Logistic regression	0.872	0.804	0.745	0.855
Test set (*n* = 22)	CatBoost	0.897	0.873	0.700	0.911
	AdaBoost	0.767	0.845	0.550	0.911
	XGBoost	0.722	0.873	0.650	0.922
	Logistic Regression	0.775	0.755	0.650	0.778

Performance of the four machine learning models in predicting cognitive decline (≥3 points of changes in the total scores of K-MMSE for 9 months) was evaluated using area under the curve (AUC), accuracy, sensitivity, and specificity scores in training (*n* = 87) and test (*n* = 22) sets.

AdaBoost, Adaptive Boosting; AUC, area under the receiver operating characteristic curve; CatBoost, Categorical Boosting; XGBoost, Extreme Gradient Boosting.

Mean AUC of the other three models ranked in the following descending order: XGBoost, 0.950; AdaBoost, 0.924; and logistic regression, 0.872. XGBoost algorithm achieved an accuracy of 0.880, a sensitivity of 0.836, and a specificity of 0.918, demonstrating the highest specificity score among the four models. AdaBoost algorithm achieved an accuracy of 0.838, a sensitivity of 0.855, and a specificity of 0.823. Logistic regression algorithm achieved an accuracy of 0.804, a sensitivity of 0.745, and a specificity of 0.855 (Table 3).

### 3.3 Classification of cognitive decliners in the test set

**Figure 3:**
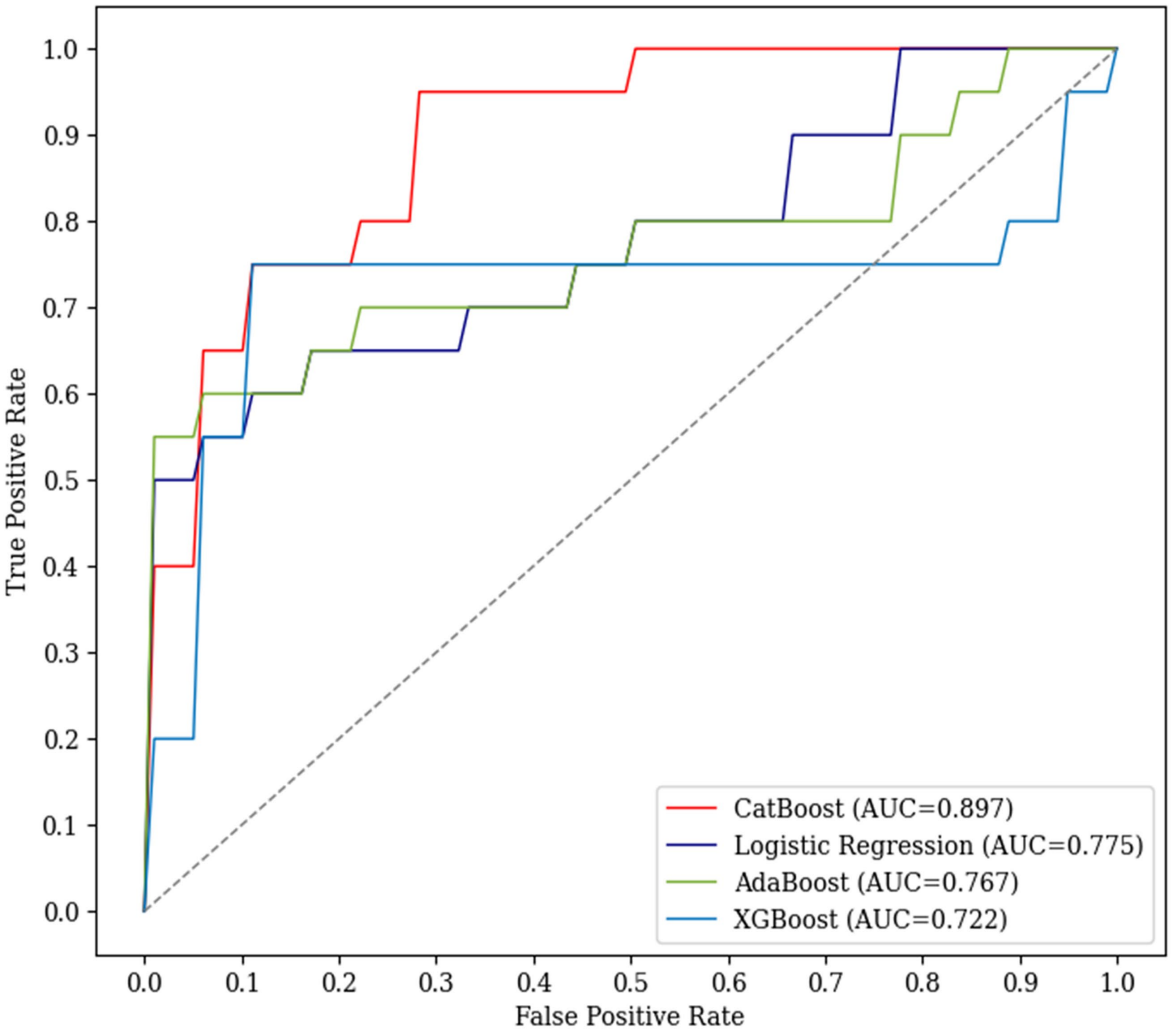
Performance of the four machine learning models in predicting cognitive decline. The ROC curve indicates AUC of the four machine learning models in predicting cognitive decline (≥3 points of changes in the total scores of K-MMSE over 9 months) of patients with PSCI in the test set. The relationship between true positive rate and false positive rate is indicated for CatBoost (red), AdaBoost (green), XGBoost (blue), and logistic regression (purple). AdaBoost, Adaptive Boosting; AUC, area under the curve; CatBoost, Categorical Boosting; K-MMSE, Korean-Mini Mental Status Examination; ROC, receiver operating characteristic; XGBoost, Extreme Gradient Boosting.

Classification results of the four machine learning models in the test set (*n* = 22) are summarized in Table 3 and Figure 3. In the test set, CatBoost outperformed the other three machine learning models in terms of AUC, accuracy, and sensitivity. It achieved a mean AUC of 0.897, an accuracy of 0.873, a sensitivity of 0.700, and a specificity of 0.911.

Mean AUC of the remaining three models ranked in the following descending order: logistic regression, 0.775; AdaBoost, 0.767; and XGBoost, 0.722. The logistic regression algorithm achieved an accuracy of 0.755, a sensitivity of 0.650, and a specificity of 0.778. The AdaBoost showed an accuracy of 0.845, a sensitivity of 0.550, and a specificity of 0.911. The XGBoost algorithm achieved an accuracy of 0.873, a sensitivity of 0.650, and a specificity of 0.922, demonstrating the highest accuracy and specificity scores among the four models (Table 2).

### 3.4 Feature importance for classification of cognitive decliners

Feature importance was determined using the SHAP methodology, with the most crucial feature ranked at the top, as depicted in Figure 2, which shows the selected input features for each model. In the CatBoost model, K-MMSE total scores, language K-MMSE subscore, initial NIHSS score, orientation to place K-MMSE subscore, memory recall K-MMSE subscore, time between stroke onset and MMSE, and lesion volume ratio were the seven most important features at baseline evaluation in predicting cognitive decliners after 9 months.

Across the four machine learning models, K-MMSE total scores, language K-MMSE subscore, and orientation to time K-MMSE subscore were the top three influential features, with higher baseline values contributing to predicting cognitive worsening after 9 months. Higher diastolic blood pressure, longer time since stroke onset, and status of current smoking contributed to predicting cognitive worsening. Patient age, initial NIHSS score, lesion volume ratio, follow-up duration, and time between stroke onset and randomization into pharmacotherapy were also included as the top seven influential factors contributing to predicting cognitive worsening across the four models.

### 3.5 Sensitivity analysis excluding the baseline K-MMSE total score

To assess the influence of baseline cognitive status on model predictions, we conducted a sensitivity analysis by removing the K-MMSE total score from the input features. As shown in Supplementary Table 2, this led to decreased performance in AUC and sensitivity across most models. For instance, in the CatBoost model, the test AUC dropped from 0.897 to 0.739, accuracy from 0.873 to 0.755, and sensitivity from 0.700 to 0.500. Despite these reductions, the models retained moderate accuracy (0.736–0.845) and specificity (0.767–0.944), indicating preserved overall discriminative ability in the test set.

Notably, the AdaBoost model demonstrated improved performance in the training set (AUC increased from 0.924 to 0.964; sensitivity from 0.855 to 0.930), and its test specificity increased from 0.911 to 0.944. This suggests that, in the absence of the baseline K-MMSE total score, the model adopted a more conservative decision threshold—prioritizing the accurate classification of non-decliners over the detection of true decliners.

SHAP analysis revealed a corresponding shift in feature importance toward clinical and imaging variables, including initial NIHSS score, diastolic blood pressure, lesion volume ratio, and the time interval from stroke onset to pharmacotherapy or cognitive assessment (Supplementary Figure 1). These findings underscore the residual predictive value of non-cognitive features, even when global baseline cognitive measures are excluded.

### 3.6 Sensitivity analysis without SMOTETomek

To evaluate the impact of resampling, we conducted a sensitivity analysis comparing model performance with and without the application of SMOTETomek (Supplementary Table 3). When SMOTETomek was not applied to the training set (*n* = 87), sensitivity markedly declined across all models except AdaBoost—most notably in XGBoost and logistic regression, where test sensitivity dropped from 0.650 to 0.250. In contrast, specificity remained high (e.g., logistic regression: 0.989). These findings indicate that SMOTETomek substantially improved sensitivity, particularly for detecting the minority class, while having minimal effect on specificity. This comparison highlights the importance of resampling strategies in addressing class imbalance and enhancing model performance for detecting cognitive decline in test data.

### 3.7 Sensitivity analysis excluding education years

To evaluate the impact of cognitive reserve proxies on model performance, we conducted a sensitivity analysis by excluding education years from the input features. As shown in Supplementary Table 4, CatBoost and AdaBoost retained or improved performance in the test set despite the exclusion. CatBoost achieved higher test accuracy (from 0.873 to 0.891) and specificity (from 0.911 to 0.922), while AdaBoost maintained stable accuracy (from 0.845 to 0.882) and showed an increase in AUC (from 0.767 to 0.875). In contrast, performance declined in XGBoost and logistic regression, particularly in sensitivity, suggesting greater dependence on education-related input.

SHAP analysis (Figure 2 and Supplementary Figure 2) further supported these findings. Although education years contributed modestly to prediction, their exclusion did not substantially affect the relative importance of key features such as baseline K-MMSE subscores, NIHSS score, diastolic blood pressure, lesion volume ratio, and stroke-to-assessment intervals. These findings suggest that the high-performing models relied primarily on cognitive and clinical variables, reinforcing their robustness in predicting cognitive decline independent of educational attainment.

## 4 Discussion

This study presents four machine learning models that use clinical and imaging data to predict patients with PSCI at high risk of cerebral hemorrhage, who are likely to experience cognitive decline within 14 months after stroke onset, which is a 9-month follow-up. Specifically, CatBoost demonstrated the highest performance in terms of AUC, accuracy, and sensitivity in training and test sets. The most influential factors for predicting cognitive decline were higher baseline K-MMSE scores (total, language, orientation to place, and recall), a longer interval between stroke onset and baseline MMSE and initial NIHSS scores, and lesion volume ratio in CatBoost. Cognitive decliners who deteriorated after 9 months (mean time since stroke, 14.0 months) had a longer interval between stroke onset and pharmacotherapy, with trends of longer duration between stroke onset and MMSE, younger age, more education, and lower LDL and HDL levels, than non-decliners who showed deterioration earlier at baseline (mean time since stroke, 4.7 months). Moreover, cognitive decliners exhibited higher baseline K-MMSE total scores and subscores compared to non-decliners.

High AUC and accuracy of our CatBoost model emphasize its reliable prediction of cognitive decline in patients with PSCI and high-risk cerebral hemorrhage. Considering typical machine learning models that predict PSCI at a single time point and focus on poststroke functional outcomes, our model excelled in predicting PSCI worsening after 9 months. Previous machine learning models demonstrated comparable predictive performance ranges, with an AUC of 0.80–0.91, an accuracy of 0.74–0.80, a sensitivity of 0.70–0.90, and a specificity of 0.68–0.82 (20, 35–38). In classifying cognitive decliners, boosting models excelled by combining weak learners and preventing overfitting through hyperparameter tuning. CatBoost effectively handled imbalanced datasets and mixed data types, achieving an AUC of 0.897 (30). XGBoost, known for its high efficiency and flexibility, achieved the highest specificity of 0.922 but the lowest AUC of 0.722 (32), AdaBoost, which combines weak classifiers (31) and logistic regression, which is valued for its interpretability (33), showed AUC scores of 0.767 and 0.775, respectively.

In CatBoost that demonstrated the highest performance, a longer interval between stroke onset and baseline MMSE and higher baseline K-MMSE scores were key predictors of cognitive worsening as determined using the SHAP methodology. Statistically, cognitive decliners had higher baseline K-MMSE scores and tended to have a longer time since stroke, be younger, and have more years of education than cognitive non-decliners. This result suggests that cognitive decliners with less baseline impairment have protective factors, such as younger age and higher education, delaying cognitive decline to the 9-month follow-up, unlike cognitive non-decliners who began deteriorating at baseline (39, 40). In AdaBoost and XGBoost, older age importantly predicted cognitive deterioration, which is supported by previous findings that advanced age increases the PSCI odds ratio from 3.5 to 9.4, alongside greater brain plaque formation and reduced blood vessel elasticity (41–43). An extended educational background, serving as a cognitive reserve, was associated with lower PSCI occurrence, lower dementia prevalence, and improved long-term survival after stroke (39, 40). Sensitivity analysis excluding education years indicated that model predictions were predominantly driven by clinically and cognitively salient features, such as baseline K-MMSE subscores, NIHSS scores, and lesion characteristics, rather than cognitive reserve proxies. This suggests that the model captures neurologically meaningful patterns and remains applicable across populations with varying educational backgrounds, supporting its generalizability in clinical contexts (17, 38, 44). Age and education, both strongly correlated with baseline MMSE scores, have been identified as key predictors in previous machine learning models of PSCI (20, 35). Additionally, higher baseline K-MMSE scores—particularly in the orientation and language domains—may reflect preserved cognitive integrity and serve as protective factors against subsequent decline. These domains are supported by temporoparietal and frontal cortical networks, which are commonly vulnerable to ischemic injury and play a central role in sustaining functional independence and cognitive resilience during post-stroke recovery (45).

In CatBoost, initial NIHSS scores and lesion volume ratio were important factors predicting cognitive decline at 14 months poststroke. These stroke severity indices, although not significantly different between cognitive decliners and non-decliners, importantly predicted PSCI after 9 months without a clear directionality. Higher initial NIHSS scores were associated with an increased risk of cognitive decline in patients with PSCI, particularly among those with subcortical stroke (10, 44, 46). Although the NIHSS was originally developed to quantify acute neurological deficits, elevated scores have also been linked to unfavorable long-term cognitive outcomes, especially in the presence of extensive white matter damage or impaired cerebral perfusion (47). Our finding that the lesion volume ratio (ischemic stroke volume relative to total brain volume) is a key predictor of PSCI aligns with previous research identifying both stroke volume and brain atrophy as major determinants of post-stroke cognition (45). White matter lesions have been identified as significant risk factors for PSCI, contributing to slowed processing speed and impaired executive function through disruption of the fronto-subcortical circuits (35, 37, 48). Larger subcortical infarcts can disrupt key networks involving the basal ganglia, thalamus, and prefrontal cortex—regions essential for working memory and cognitive control—and are strongly linked to PSCI, particularly in small vessel disease (37, 47).

In both the AdaBoost and XGBoost models, the time interval between stroke onset and pharmacotherapy initiation emerged as a significant predictor of cognitive deterioration. In the PICASSO trial, this interval was significantly longer among cognitive decliners than among non-decliners. The trial showed that pharmacotherapy with cilostazol or aspirin, with and without probucol reduced cardiovascular events in patients with ischemic stroke and a high risk of hemorrhage (12). Delayed treatment may compromise cerebrovascular integrity, prolong inflammation, and hinder neurovascular repair, thereby increasing the risk of post-stroke cognitive deterioration (49). These findings suggest that early pharmacologic intervention may play a protective role against cognitive deterioration by mitigating vascular events and supporting recovery mechanisms (50). Consistent with this, diastolic blood pressure and current smoking status were within the top five predictors of cognitive decline in AdaBoost, XGBoost, and logistic regression, which is supported by previous findings that PSCI is closely associated with vascular risk factors such as hypertension, smoking history, diabetes mellitus, and heart disease, all affecting inflammation and cerebral perfusion (20, 38, 51). A trend toward lower LDL and HDL levels, observed in cognitive decliners, suggests disturbed neural maintenance and antioxidant effects underlying cognitive worsening in patients with PSCI (38, 52).

These findings suggest that the model captures not only statistical associations but also underlying pathophysiological mechanisms contributing to post-stroke cognitive decline. The neurobiological relevance of prioritized features supports their clinical utility and mechanistic validity in predicting cognitive trajectories. A predictive model capable of identifying patients at high risk for PSCI within the first year post-stroke may facilitate timely and personalized interventions. High-risk individuals may benefit from early initiation of tailored and intensive cognitive rehabilitation, more frequent neurocognitive monitoring (e.g., every 3–6 months), and earlier use of cognitive-enhancing pharmacologic treatments such as cholinesterase inhibitors or memantine (53, 54). Clinicians may also re-evaluate secondary prevention strategies, including stricter control of vascular risk factors and adjustment of antiplatelet regimens to minimize the risk of hemorrhagic complications (55). Early identification allows for proactive caregiver involvement and planning for personalized support services, helping families prepare for potential cognitive deterioration. Collectively, these targeted interventions may attenuate decline, reduce long-term disability, and enhance functional recovery in patients with PSCI at high risk of cognitive deterioration.

To further assess model robustness and the influence of baseline cognitive status, we conducted a sensitivity analysis excluding the K-MMSE total score. This led to a reduction in AUC and sensitivity in most models, highlighting the strong predictive weight of baseline cognition. Interestingly, AdaBoost demonstrated improved training performance and increased test specificity, indicating a more conservative classification pattern prioritizing the accurate identification of non-decliners at the cost of missing true decliners. SHAP analysis revealed a shift in feature importance toward non-cognitive variables such as NIHSS score, diastolic blood pressure, lesion volume ratio, and timing of pharmacotherapy or cognitive evaluation. These results suggest that even in the absence of global cognitive scores, meaningful clinical and imaging predictors of cognitive decline can still be identified. However, the trade-off in reduced sensitivity underscores the need to balance predictor selection with intended clinical use—particularly in early detection versus diagnostic confirmation contexts.

Several limitations should be considered when interpreting these findings. The relatively small sample size (*n* = 109) and retrospective design may limit the generalizability of the results and introduce potential selection or information biases. The small size of the test set (*n* = 22) limits the stability of performance estimates, particularly sensitivity. While internal cross-validation and standardized data collection provide some reassurance, external validation with larger, prospective, and multicenter cohorts is essential to confirm the generalizability, robustness, and clinical applicability of our model.

Additionally, class imbalance—cognitive decliners made up only 24.7% of the sample—may have contributed to reduced sensitivity. To address this, we applied SMOTETomek, which combines oversampling of the minority class with the removal of borderline majority class samples. This method was applied only to the training set to prevent data leakage and preserve the original class distribution in the test set. As shown in our sensitivity analysis (Supplementary Table 3), removing SMOTETomek led to a marked drop in sensitivity—particularly in XGBoost and logistic regression—while specificity remained high. This demonstrates its utility in improving minority class detection with minimal loss of specificity. However, synthetic sampling can introduce bias or overfitting, particularly in small datasets. To mitigate this, we used stratified k-fold cross-validation to maintain class proportions and ensure reliable performance estimates, and performed SHAP analysis to confirm the stability and clinical plausibility of feature importance. Future studies should incorporate external validation with larger and more diverse cohorts. In addition to SMOTETomek, methods such as bootstrap resampling and integration of multimodal clinical and imaging data may further enhance model performance.

Furthermore, the operational definition of cognitive decline as a ≥ 3-point decrease in K-MMSE scores over 9 months, while consistent with prior studies, may not fully capture the multidimensional nature of cognitive deterioration. Incorporating clinical assessments and a broader range of neuropsychological tools would provide a more comprehensive evaluation framework. Future research should also consider extending the follow-up period to better understand the long-term trajectory of PSCI beyond 9 months. Our sensitivity analysis demonstrated that excluding the baseline K-MMSE total score resulted in a reduction in AUC and sensitivity, highlighting its critical role in the early detection of cognitive decline. Nonetheless, the increased relative importance of non-cognitive features—such as lesion volume, blood pressure, stroke severity, stroke duration, and timing of pharmacotherapy—suggests that models omitting global cognitive scores may still capture clinically relevant predictors. Future studies should consider excluding baseline cognitive scores to better delineate the contributions of alternative features, while carefully addressing the inherent trade-off in predictive sensitivity.

In conclusion, machine learning models, particularly the CatBoost algorithm, may reliably predict patients with PSCI with high-risk cerebral hemorrhage, who may experience cognitive decline within 14 months after stroke onset. According to SHAP and statistical analyses, cognitive decliners had protective factors of younger age and extended education, which delayed deterioration till the 9-month follow-up, compared with cognitive non-decliners who showed cognitive worsening earlier at baseline. A longer interval between stroke onset and pharmacotherapy, along with smoking status and cholesterol levels, may contribute to predicting cognitive decline as risk factors.

## Data Availability

The raw data supporting the conclusions of this article will be made available by the authors, without undue reservation.

## References

[1] Gorelick PB. The global burden of stroke: persistent and disabling. Lancet Neurol. (2019) 18:417–8. doi: 10.1016/S1474-4422(19)30030-4, PMID: 30871943

[2] Jokinen H Melkas S Ylikoski R Pohjasvaara T Kaste M Erkinjuntti T. Post-stroke cognitive impairment is common even after successful clinical recovery. Eur J Neurol. (2015) 22:1288–94. doi: 10.1111/ene.12743, PMID: 26040251

[3] Pendlebury ST Rothwell PM. Prevalence, incidence, and factors associated with pre-stroke and post-stroke dementia: a systematic review and meta-analysis. Lancet Neurol. (2009) 8:1006–18. doi: 10.1016/S1474-4422(09)70236-4, PMID: 19782001

[4] Melkas S Oksala NK Jokinen H Pohjasvaara T Vataja R Oksala A. Poststroke dementia predicts poor survival in long-term follow-up: influence of prestroke cognitive decline and previous stroke. J Neurol Neurosurg Psychiatry. (2009) 80:865–70. doi: 10.1136/jnnp.2008.166603, PMID: 19240049

[5] Nys G Van Zandvoort M De Kort P Van Der Worp H Jansen B Algra A. The prognostic value of domain-specific cognitive abilities in acute first-ever stroke. Neurology. (2005) 64:821–7. doi: 10.1212/01.WNL.0000152984.28420.5A 15753416

[6] Pendlebury ST. Dementia in patients hospitalized with stroke: rates, time course, and clinico-pathologic factors. Int J Stroke. (2012) 7:570–81. doi: 10.1111/j.1747-4949.2012.00837.x, PMID: 22781124

[7] Yu K-H Cho S-J Oh MS Jung S Lee J-H Shin J-H. Cognitive impairment evaluated with vascular cognitive impairment harmonization standards in a multicenter prospective stroke cohort in Korea. Stroke. (2013) 44:786–8. doi: 10.1161/STROKEAHA.112.668343, PMID: 23271507

[8] Martinez-Ramirez S Greenberg SM Viswanathan A. Cerebral microbleeds: overview and implications in cognitive impairment. Alzheimers Res Ther. (2014) 6:1–7. doi: 10.1186/alzrt263, PMID: 24987468 PMC4075149

[9] Yamada M. Cerebral amyloid angiopathy: emerging concepts. J Stroke. (2015) 17:17–30. doi: 10.5853/jos.2015.17.1.17, PMID: 25692104 PMC4325636

[10] Diener H-C Bogousslavsky J Brass LM Cimminiello C Csiba L Kaste M. Aspirin and clopidogrel compared with clopidogrel alone after recent ischaemic stroke or transient ischaemic attack in high-risk patients (MATCH): randomised, double-blind, placebo-controlled trial. Lancet. (2004) 364:331–7. doi: 10.1016/S0140-6736(04)16721-4 15276392

[11] Johnston SC Amarenco P Albers GW Denison H Easton JD Evans SR. Ticagrelor versus aspirin in acute stroke or transient ischemic attack. N Engl J Med. (2016) 375:35–43. doi: 10.1056/NEJMoa1603060, PMID: 27160892

[12] Kim BJ Lee EJ Kwon SU Park JH Kim YJ Hong KS. Prevention of cardiovascular events in Asian patients with ischaemic stroke at high risk of cerebral haemorrhage (PICASSO): a multicentre, randomised controlled trial. Lancet Neurol. (2018) 17:509–18. doi: 10.1016/S1474-4422(18)30128-5, PMID: 29778364

[13] Shinohara Y Katayama Y Uchiyama S Yamaguchi T Handa S Matsuoka K. Cilostazol for prevention of secondary stroke (CSPS 2): an aspirin-controlled, double-blind, randomised non-inferiority trial. Lancet Neurol. (2010) 9:959–68. doi: 10.1016/S1474-4422(10)70198-8, PMID: 20833591

[14] Tardif J-C Côté G Lespérance J Bourassa M Lambert J Doucet S. Probucol and multivitamins in the prevention of restenosis after coronary angioplasty. N Engl J Med. (1997) 337:365–72. doi: 10.1056/NEJM199708073370601, PMID: 9241125

[15] Kim BJ Kim JS. Ischemic stroke subtype classification: an Asian viewpoint. J Stroke. (2014) 16:8–17. doi: 10.5853/jos.2014.16.1.8, PMID: 24741560 PMC3961817

[16] Qureshi AI Mendelow AD Hanley DF. Intracerebral haemorrhage. Lancet. (2009) 373:1632–44. doi: 10.1016/S0140-6736(09)60371-8, PMID: 19427958 PMC3138486

[17] Filler J Georgakis MK Dichgans M. Risk factors for cognitive impairment and dementia after stroke: a systematic review and meta-analysis. Lancet Healthy Longev. (2024) 5:e31–44. doi: 10.1016/S2666-7568(23)00217-9, PMID: 38101426

[18] Mijajlović MD Pavlović A Brainin M Heiss W-D Quinn TJ Ihle-Hansen HB. Post-stroke dementia–a comprehensive review. BMC Med. (2017) 15:1–12. doi: 10.1186/s12916-017-0779-7 28095900 PMC5241961

[19] Murphy TH Corbett D. Plasticity during stroke recovery: from synapse to behaviour. Nat Rev Neurosci. (2009) 10:861–72. doi: 10.1038/nrn2735, PMID: 19888284

[20] Li X Chen Z Jiao H Wang B Yin H Chen L. Machine learning in the prediction of post-stroke cognitive impairment: a systematic review and meta-analysis. Front Neurol. (2023) 14:1211733. doi: 10.3389/fneur.2023.1211733, PMID: 37602236 PMC10434510

[21] Rasquin S Verhey F Van Oostenbrugge R Lousberg R Lodder J. Demographic and CT scan features related to cognitive impairment in the first year after stroke. J Neurol Neurosurg Psychiatry. (2004) 75:1562–7. doi: 10.1136/jnnp.2003.024190, PMID: 15489388 PMC1738816

[22] Yu K-H Hong K-S Oh M-S Lee J Lee JS Kwon SU. Design and rationale for a cognitive outcome substudy in ischemic stroke patients with high risk of cerebral hemorrhage. J Stroke Cerebrovasc Dis. (2016) 25:2061–6. doi: 10.1016/j.jstrokecerebrovasdis.2016.04.028, PMID: 27263034

[23] Kang Y Na D-L Hahn S. A validity study on the Korean Mini-mental state examination (K-MMSE) in dementia patients. J Korean Neurol Assoc. (1997) 15:300–8.

[24] Kwah LK Diong J. National institutes of health stroke scale (NIHSS). J Physiother. (2014) 60:12. doi: 10.1016/j.jphys.2013.12.012, PMID: 24856948

[25] Fazekas F Chawluk JB Alavi A Hurtig HI Zimmerman RA. MR signal abnormalities at 1.5 T in Alzheimer's dementia and normal aging. Am J Roentgenol. (1987) 149:351–6. doi: 10.2214/ajr.149.2.351, PMID: 3496763

[26] Schmidt P. Wink L. (2017) LST: a lesion segmentation tool for SPM. Manual. Version 2.0. Jülich (Germany): Institute of Neuroscience and Medicine, Research Center Jülich.

[27] Hensel A Angermeyer MC Riedel-Heller SG. Measuring cognitive change in older adults: reliable change indices for the Mini-mental state examination. J Neurol Neurosurg Psychiatry. (2007) 78:1298–303. doi: 10.1136/jnnp.2006.109074, PMID: 17442763 PMC2095596

[28] Tombaugh TN. Test-retest reliable coefficients and 5-year change scores for the MMSE and 3MS. Arch Clin Neuropsychol. (2005) 20:485–503. doi: 10.1016/j.acn.2004.11.004, PMID: 15896562

[29] Wang Z Wu C Zheng K Niu X Wang X. Smotetomek-based resampling for personality recognition. IEEE Access. (2019) 7:129678–89. doi: 10.1109/ACCESS.2019.2940061

[30] Prokhorenkova L Gusev G Vorobev A Dorogush AV Gulin A. CatBoost: unbiased boosting with categorical features In: Bengio S, Wallach HM, Larochelle H, Grauman K, Cesa‑Bianchi N, Garnett R, editors. Advances in neural information processing systems. Vol. 31. Curran Associates, Inc. (2018) p. 6639–49.

[31] Freund Y Schapire RE. A decision-theoretic generalization of on-line learning and an application to boosting. J Comput Syst Sci. (1997) 55:119–39. doi: 10.1006/jcss.1997.1504

[32] Chen T. Guestrin C. (2016). Xgboost: a scalable tree boosting system, in: Proceedings of the 22nd acm sigkdd international conference on knowledge discovery and data mining, 785–794.

[33] Christodoulou E Ma J Collins GS Steyerberg EW Verbakel JY Van Calster B. A systematic review shows no performance benefit of machine learning over logistic regression for clinical prediction models. J Clin Epidemiol. (2019) 110:12–22. doi: 10.1016/j.jclinepi.2019.02.004, PMID: 30763612

[34] Mangalathu S Hwang S-H Jeon J-S. Failure mode and effects analysis of RC members based on machine-learning-based SHapley additive exPlanations (SHAP) approach. Eng Struct. (2020) 219:110927. doi: 10.1016/j.engstruct.2020.110927

[35] Chander RJ Lam BYK Lin X Ng AYT Wong APL Mok VCT. Development and validation of a risk score (CHANGE) for cognitive impairment after ischemic stroke. Sci Rep. (2017) 7:12441. doi: 10.1038/s41598-017-12755-z, PMID: 28963553 PMC5622067

[36] Drozdowska BA Mcgill K Mckay M Bartlam R Langhorne P Quinn TJ. Prognostic rules for predicting cognitive syndromes following stroke: a systematic review. Eur Stroke J. (2021) 6:18–27. doi: 10.1177/2396987321997045, PMID: 33817331 PMC7995322

[37] Kandiah N Wiryasaputra L Narasimhalu K Karandikar A Marmin M Chua EV. Frontal subcortical ischemia is crucial for post stroke cognitive impairment. J Neurol Sci. (2011) 309:92–5. doi: 10.1016/j.jns.2011.07.013, PMID: 21807379

[38] Lee M Yeo N-Y Ahn H-J Lim J-S Kim Y Lee S-H. Prediction of post-stroke cognitive impairment after acute ischemic stroke using machine learning. Alzheimers Res Ther. (2023) 15:147. doi: 10.1186/s13195-023-01289-4, PMID: 37653560 PMC10468853

[39] Brayne C. Ince P.G. Keage H.A. Mckeith I.G. Matthews F.E. Polvikoski T.. (2010) Education, the brain and dementia: neuroprotection or compensation? EClipSE Collaborative Members Brain 133 2210–2216. doi: 10.1093/brain/awq185, PMID: 20826429

[40] Ojala-Oksala J Jokinen H Kopsi V Lehtonen K Luukkonen L Paukkunen A. Educational history is an independent predictor of cognitive deficits and long-term survival in postacute patients with mild to moderate ischemic stroke. Stroke. (2012) 43:2931–5. doi: 10.1161/STROKEAHA.112.667618, PMID: 22935400

[41] Brainin M Tuomilehto J Heiss WD Bornstein NM Bath PM Teuschl Y. Post-stroke cognitive decline: an update and perspectives for clinical research. Eur J Neurol. (2015) 22:229–38. doi: 10.1111/ene.12626 25492161

[42] Rasquin SM Lodder J Ponds RW Winkens I Jolles J Verhey FR. Cognitive functioning after stroke: a one-year follow-up study. Dementia Geriatr Cogn Disord. (2004) 18:138–44. doi: 10.1159/000079193, PMID: 15211068

[43] Zimmerman B Rypma B Gratton G Fabiani M. Age-related changes in cerebrovascular health and their effects on neural function and cognition: a comprehensive review. Psychophysiology. (2021) 58:e13796. doi: 10.1111/psyp.13796, PMID: 33728712 PMC8244108

[44] Glymour MM Berkman LF Ertel KA Fay ME Glass TA Furie KL. Lesion characteristics, NIH stroke scale, and functional recovery after stroke. Am J Phys Med Rehabil. (2007) 86:725–33. doi: 10.1097/PHM.0b013e31813e0a32, PMID: 17709996

[45] Puy L Barbay M Roussel M Canaple S Lamy C Arnoux A. Neuroimaging determinants of poststroke cognitive performance: the GRECogVASC study. Stroke. (2018) 49:2666–73. doi: 10.1161/STROKEAHA.118.021981 30355190

[46] Liu Z Liu Y Tu X Shen H Qiu H Chen H. High serum levels of malondialdehyde and 8-OHdG are both associated with early cognitive impairment in patients with acute ischaemic stroke. Sci Rep. (2017) 7:9493. doi: 10.1038/s41598-017-09988-3, PMID: 28842715 PMC5573400

[47] Munsch F Sagnier S Asselineau J Bigourdan A Guttmann CR Debruxelles S. Stroke location is an independent predictor of cognitive outcome. Stroke. (2016) 47:66–73. doi: 10.1161/STROKEAHA.115.011242, PMID: 26585396

[48] Kandiah N Chander RJ Lin X Ng A Poh YY Cheong CY. Cognitive impairment after mild stroke: development and validation of the SIGNAL2 risk score. J Alzheimers Dis. (2016) 49:1169–77. doi: 10.3233/JAD-150736, PMID: 26599056

[49] Iadecola C Anrather J. The immunology of stroke: from mechanisms to translation. Nat Med. (2011) 17:796–808. doi: 10.1038/nm.2399, PMID: 21738161 PMC3137275

[50] Moskowitz MA Lo EH Iadecola C. The science of stroke: mechanisms in search of treatments. Neuron. (2010) 67:181–98. doi: 10.1016/j.neuron.2010.07.002, PMID: 20670828 PMC2957363

[51] Sahathevan R Brodtmann A Donnan GA. Dementia, stroke, and vascular risk factors; a review. Int J Stroke. (2012) 7:61–73. doi: 10.1111/j.1747-4949.2011.00731.x, PMID: 22188853

[52] Kim KY Shin KY Chang K-A. Potential biomarkers for post-stroke cognitive impairment: a systematic review and meta-analysis. Int J Mol Sci. (2022) 23:602. doi: 10.3390/ijms23020602, PMID: 35054785 PMC8775398

[53] Cicerone KD Langenbahn DM Braden C Malec JF Kalmar K Fraas M. Evidence-based cognitive rehabilitation: updated review of the literature from 2003 through 2008. Arch Phys Med Rehabil. (2011) 92:519–30. doi: 10.1016/j.apmr.2010.11.015, PMID: 21440699

[54] Román GC Sachdev P Royall DR Bullock RA Orgogozo J-M López-Pousa S. Vascular cognitive disorder: a new diagnostic category updating vascular cognitive impairment and vascular dementia. J Neurol Sci. (2004) 226:81–7. doi: 10.1016/j.jns.2004.09.016, PMID: 15537526

[55] Kernan WN Ovbiagele B Black HR Bravata DM Chimowitz MI Ezekowitz MD. Guidelines for the prevention of stroke in patients with stroke and transient ischemic attack: a guideline for healthcare professionals from the American Heart Association/American Stroke Association. Stroke. (2014) 45:2160–236. doi: 10.1161/STR.0000000000000024, PMID: 24788967

