# Supplementary material for: Predicting cognitive decline in cognitively impaired patients with ischemic stroke with high risk of cerebral hemorrhage: a machine learning approach
Source: Front Neurol. 2025 Jul 25;16:1569073. doi: 10.3389/fneur.2025.1569073 (PMC12333937; doi:10.3389/fneur.2025.1569073)
Supplement: Supplementary file 1 [file Data_Sheet_1.docx]

**SUPPLEMENTARY MATERIAL**

Predicting cognitive decline in cognitively impaired patients with ischemic stroke with high risk of cerebral hemorrhage: a machine learning approach

**Supplementary Table 1.** Summary of the machine learning workflow.

| **Step** | **Description** | **Tool / Package (Version)** | **Output** |
| --- | --- | --- | --- |
| 1. Data Input | Load clinical and imaging data (demographics, NIHSS & K-MMSE scores, lesion volume) | pandas | Raw dataset (n = 109) |
| 2. Feature Processing | Quantify lesion volume using LST (SPM12); compute lesion volume ratio; standardize continuous variables | scikit-learn (v1.1.3),  LST (SPM12) | Standardized features; numeric lesion features |
| 3. Data Labeling | Define cognitive decline as a ≥3-point decrease in K-MMSE scores over 9 months | Manual rule-based classification | Binary labels (decliner vs. non-decliner) |
| 4. Train/Test Split | Perform stratified 80/20 train/test split by outcome label (random_state = 1004) | scikit-learn (v1.1.3) | Training set (n = 87), Test set (n = 22) |
| 5. Resampling | Apply SMOTETomek to the training set only (sampling_strategy = 0.9, random_state = 42) | imbalanced-learn (v0.10.1) | Balanced training set |
| 6. Feature Selection | Use SHAP to identify high-impact features and exclude low-impact features; generate summary plots | SHAP (v0.44.1) | Reduced feature set |
| 7. Model Training | Train models with 5-fold StratifiedKFold cross-validation (random_state = 1004) | scikit-learn (logistic regression, AdaBoost (v1.1.3), CatBoost (v1.1.1), XGBoost (v1.4.2) | Trained classifiers |
| 8. Hyperparameter Tuning | Optimize hyperparameters using GridSearchCV for AdaBoost/XGBoost; RandomizedSearchCV (n_iter = 10) for logistic regression/CatBoost; (random_state = 1004) | scikit-learn (v1.1.3) | Optimal hyperparameters |
| 9. Model Evaluation | Compute AUC, accuracy, sensitivity, and specificity; optimize the threshold using the Youden Index | scikit-learn (metrics), matplotlib | Performance metrics |
| 10. Model Validation | Evaluate the final model on the independent test set, preserving the original class distribution | scikit-learn (v1.1.3) | Test set results |

This table outlines the end-to-end machine learning pipeline implemented in the study. The workflow comprised data preprocessing, lesion quantification, outcome labeling, resampling, feature selection, model training, hyperparameter optimization, and performance evaluation. Model performance was assessed on a stratified held-out test set that preserved the original class distribution. To ensure reproducibility, all stochastic procedures—including data partitioning, resampling, cross-validation, and model initialization—were performed using fixed random seeds. Software tools and package versions are reported to facilitate transparency and replication.

**Abbreviations:** AdaBoost, Adaptive Boosting; AUC, area under the receiver operating characteristic curve; CatBoost, Categorical Boosting; CV, cross-validation; K-MMSE, Korean Mini-Mental State Examination; LST, Lesion Segmentation Tool; NIHSS, National Institutes of Health Stroke Scale; ROC, receiver operating characteristic curve; SHAP, SHapley Additive exPlanations; SMOTETomek, Synthetic Minority Over-sampling Technique combined with Tomek links; SPM, Statistical Parametric Mapping; XGBoost, Extreme Gradient Boosting.

**Supplementary Table 2**. Performance of the machine learning models excluding the baseline K-MMSE total score.

| **Dataset** | **Model** | **AUC** | **Accuracy** | **Sensitivity** | **Specificity** |
| --- | --- | --- | --- | --- | --- |
| Training set  (n = 87) | CatBoost | 0.945 | 0.861 | 0.907 | 0.819 |
|  | AdaBoost | 0.964 | 0.924 | 0.930 | 0.918 |
|  | XGBoost | 0.931 | 0.779 | 0.638 | 0.904 |
|  | Logistic Regression | 0.852 | 0.781 | 0.714 | 0.838 |
| Test set  (n = 22) | CatBoost | 0.739 | 0.755 | 0.500 | 0.811 |
|  | AdaBoost | 0.694 | 0.845 | 0.400 | 0.944 |
|  | XGBoost | 0.644 | 0.755 | 0.300 | 0.855 |
|  | Logistic Regression | 0.750 | 0.736 | 0.600 | 0.767 |

Performance of four machine learning models (CatBoost, AdaBoost, XGBoost, and logistic regression) evaluated using AUC, accuracy, sensitivity, and specificity on training (n = 87) and test (n = 22) datasets after excluding the baseline K-MMSE total score from input features. All other clinical variables, imaging features, and K-MMSE subscores were included. Cognitive decline was defined as a ≥3-point decrease in total K-MMSE score over 9 months.

**Abbreviations:** AdaBoost, Adaptive Boosting; AUC, area under the receiver operating characteristic curve; CatBoost, Categorical Boosting; K-MMSE, Korean Mini-Mental State Examination; XGBoost, Extreme Gradient Boosting

**Supplementary Table 3**. Performance of the machine learning models evaluated without applying SMOTETomek

| **Dataset** | **Model** | **AUC** | **Accuracy** | **Sensitivity** | **Specificity** |
| --- | --- | --- | --- | --- | --- |
| Training set  (n = 87) | CatBoost | 0.782 | 0.804 | 0.530 | 0.904 |
|  | AdaBoost | 0.790 | 0.793 | 0.490 | 0.904 |
|  | XGBoost | 0.801 | 0.781 | 0.210 | 0.985 |
|  | Logistic Regression | 0.819 | 0.851 | 0.490 | 0.983 |
| Test set  (n = 22) | CatBoost | 0.850 | 0.909 | 0.600 | 0.978 |
|  | AdaBoost | 0.792 | 0.891 | 0.700 | 0.933 |
|  | XGBoost | 0.807 | 0.845 | 0.250 | 0.978 |
|  | Logistic Regression | 0.717 | 0.855 | 0.250 | 0.989 |

Performance metrics (AUC, accuracy, sensitivity, and specificity) of four machine learning models—CatBoost, AdaBoost, XGBoost, and logistic regression—for predicting cognitive decline, defined as a ≥3-point decrease in total K-MMSE score over 9 months. Models were evaluated on training (n = 87) and test (n = 22) sets without applying SMOTETomek, a technique that combines SMOTE-based oversampling and Tomek Links undersampling to address class imbalance.

**Abbreviations:** AdaBoost, Adaptive Boosting; AUC, area under the receiver operating characteristic curve; CatBoost, Categorical Boosting; SMOTETomek, Synthetic Minority Over-sampling Technique with Tomek Links; XGBoost, Extreme Gradient Boosting

**Supplementary Table 4**. Performance of the machine learning models excluding education years.

| **Dataset** | **Model** | **AUC** | **Accuracy** | **Sensitivity** | **Specificity** |
| --- | --- | --- | --- | --- | --- |
| Training set  (n = 87) | CatBoost | 0.917 | 0.884 | 0.877 | 0.888 |
|  | AdaBoost | 0.944 | 0.887 | 0.889 | 0.886 |
|  | XGBoost | 0.948 | 0.878 | 0.814 | 0.936 |
|  | Logistic Regression | 0.871 | 0.783 | 0.758 | 0.800 |
| Test set  (n = 22) | CatBoost | 0.889 | 0.891 | 0.750 | 0.922 |
|  | AdaBoost | 0.875 | 0.882 | 0.500 | 0.967 |
|  | XGBoost | 0.747 | 0.800 | 0.450 | 0.878 |
|  | Logistic Regression | 0.814 | 0.737 | 0.600 | 0.767 |

Performance of four machine learning models (CatBoost, AdaBoost, XGBoost, and logistic regression) evaluated using AUC, accuracy, sensitivity, and specificity on training (n = 87) and test (n = 22) datasets after excluding the education years from input features. All other clinical variables and imaging features were included. Cognitive decline was defined as a ≥3-point decrease in total K-MMSE score over 9 months.

**Abbreviations:** AdaBoost, Adaptive Boosting; AUC, area under the receiver operating characteristic curve; CatBoost, Categorical Boosting; K-MMSE, Korean Mini-Mental State Examination; XGBoost, Extreme Gradient Boosting

**Supplementary Figure 1.** SHAP summary plots after excluding the baseline K-MMSE total score.


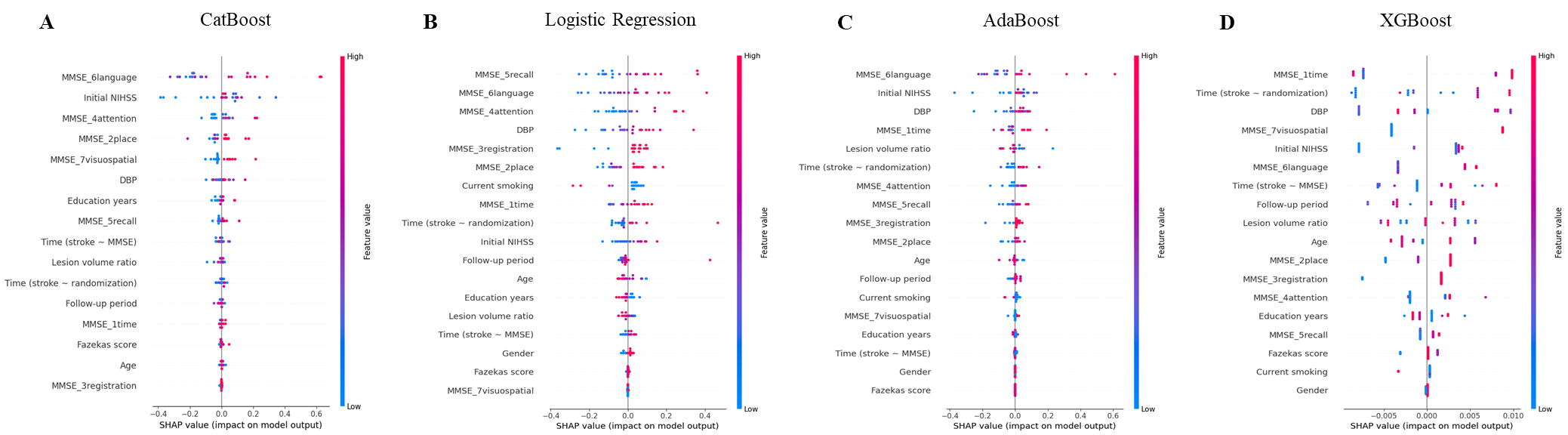


Feature importance plots from four models—(A) CatBoost, (B) Logistic Regression, (C) AdaBoost, and (D) XGBoost—after removing the baseline K-MMSE total score from input features. Each dot represents a SHAP value for a given feature and patient. Red indicates higher feature values; blue indicates lower values.

**Abbreviations:** AdaBoost, Adaptive Boosting; CatBoost, Categorical Boosting; DBP, Diastolic Blood Pressure; K-MMSE, Korean-Mini Mental Status Examination; PSCI, Poststroke Cognitive Impairment; SHAP, SHapley Additive exPlanations; XGBoost, Extreme Gradient Boosting.

**Supplementary Figure 2.** SHAP summary plots after excluding education years


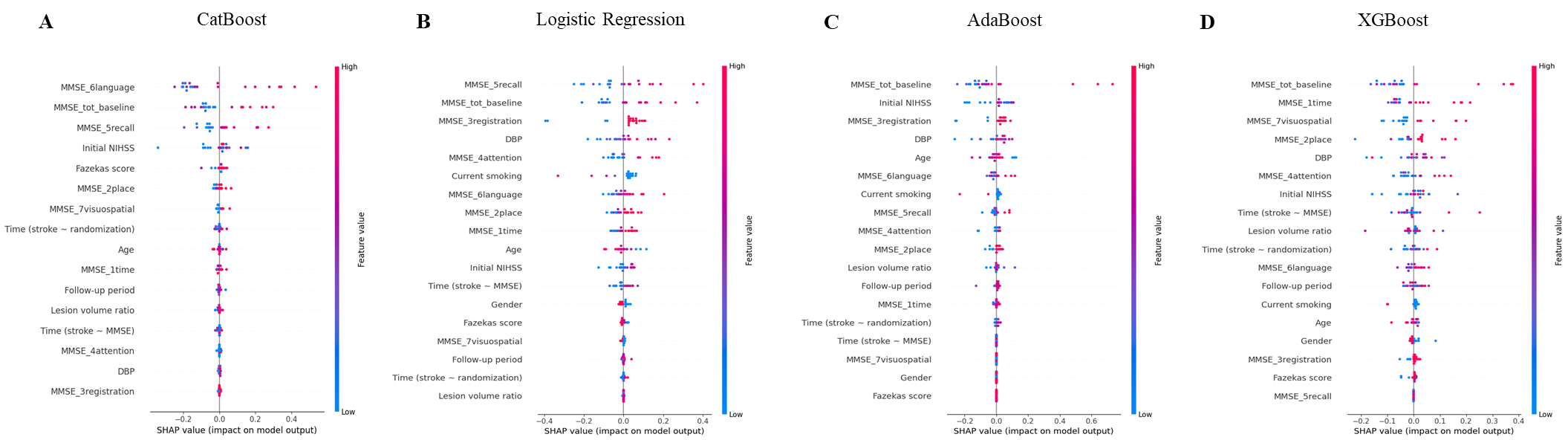


Feature importance plots from four models—(A) CatBoost, (B) Logistic Regression, (C) AdaBoost, and (D) XGBoost—after removing education years from input features. Each dot represents a SHAP value for a given feature and patient. Red indicates higher feature values; blue indicates lower values.

Abbreviations: AdaBoost, Adaptive Boosting; CatBoost, Categorical Boosting; DBP, Diastolic Blood Pressure; K-MMSE, Korean-Mini Mental Status Examination; PSCI, Poststroke Cognitive Impairment; SHAP, SHapley Additive exPlanations; XGBoost, Extreme Gradient Boosting.
