# Supplementary material for: Predicting cognitive decline in cognitively impaired patients with ischemic stroke with high risk of cerebral hemorrhage: a machine learning approach
Source: Front Neurol. 2025 Jul 25;16:1569073. doi: 10.3389/fneur.2025.1569073 (PMC12333937; doi:10.3389/fneur.2025.1569073)
Supplement: Supplementary file 2 [file Table_1.docx]

**SUPPLEMENTARY MATERIAL**

Predicting cognitive decline in cognitively impaired patients with ischemic stroke with high risk of cerebral hemorrhage: a machine learning approach

**List of Institutional Review Boards (IRBs) for the PICASSO Study**

| **IRB/Institution Name** | **Affiliation/Location** |
| --- | --- |
| Pamela Youde Nethersole Eastern Hospital | Hong Kong, China |
| Queen Elizabeth Hospital | Hong Kong, China |
| United Christian Hospital | Hong Kong, China |
| Prince of Wales Hospital | Shatin, NT, Hong Kong, China |
| Kosin University Gospel Hospital | Busan, Korea, Republic of |
| Inje University Pusan Paik Hospital | Busan, Korea, Republic of |
| Pusan National University Hospital | Busan, Korea, Republic of |
| Dong-A University Hospital | Busan, Korea, Republic of |
| Keimyung University Dongsan Center | Daegu, Korea, Republic of |
| Daegu Fatima Hospital | Daegu, Korea, Republic of |
| Eulji University Hospital | Daejeon, Korea, Republic of |
| Dongsan Medical Center | Daegu, Korea, Republic of |
| Kyungpook National University Hospital | Daegu, Korea, Republic of |
| Yeungnam University Medical Center | Daegu, Korea, Republic of |
| Daegu Catholic University Hospital | Daegu, Korea, Republic of |
| Chungnam National University Hospital | Daejeon, Korea, Republic of |
| Daejeon St. Mary's Hospital | Daejeon, Korea, Republic of |
| Chosun University Hospital | Gwangju, Korea, Republic of |
| Chonnam National University Hospital | Gwangju, Korea, Republic of |
| Gachon University Gil Hospital | Incheon, Korea, Republic of |
| Inha University Hospital | Incheon, Korea, Republic of |
| Wallace Memorial Baptist Hospital | Busan, Korea, Republic of |
| National Medical Center | Seoul, Korea, Republic of |
| Kangbuk Samsung Hospital | Seoul, Korea, Republic of |
| Severance Hospital | Seoul, Korea, Republic of |
| Kyung Hee University Medical Center | Seoul, Korea, Republic of |
| Hanyang University Medical Center | Seoul, Korea, Republic of |
| Kangdong Sacred Heart Hospital | Seoul, Korea, Republic of |
| Gangnam Severance Hospital | Seoul, Korea, Republic of |
| Seoul Medical Center | Seoul, Korea, Republic of |
| Korea University Anam Hospital | Seoul, Korea, Republic of |
| Seoul St. Mary's Hospital | Seoul, Korea, Republic of |
| Asan Medical Center | Seoul, Korea, Republic of |
| Inje University Sanggye Paik Hospital | Seoul, Korea, Republic of |
| Konkuk University Hospital | Seoul, Korea, Republic of |
| St. Mary's Hospital | Seoul, Korea, Republic of |
| Hangang Sacred Heart Hospital | Seoul, Korea, Republic of |
| Kangnam Sacred Heart Hospital | Seoul, Korea, Republic of |
| Korea University Guro Hospital | Seoul, Korea, Republic of |
| Seoul National University Borame Hospital | Seoul, Korea, Republic of |
| Ewha Womans University Medical Center | Seoul, Korea, Republic of |
| Eulji Hospital | Seoul, Korea, Republic of |
| Chung-Ang University Medical Center | Seoul, Korea, Republic of |
| Seoul National University Hospital | Seoul, Korea, Republic of |
| Soonchunhyang University Hospital | Seoul, Korea, Republic of |
| Ulsan University Hospital | Ulsan, Korea, Republic of |
| Chungbuk National University Hospital | Cheongju, Korea, Republic of |
| Soonchunhyang University Cheonan Hospital | Cheonan, Korea, Republic of |
| Wonju Christian Hospital | Wonju, Korea, Republic of |
| Kwandong University Myongji Hospital | Goyang, Korea, Republic of |
| Gyeongsang National University Hospital | Jinju, Korea, Republic of |
| Korea University Ansan Hospital | Ansan, Korea, Republic of |
| Soonchunhyang University Bucheon Hospital | Bucheon, Korea, Republic of |
| National Health Insurance Ilsan Hospital | Goyang, Korea, Republic of |
| Inje University Ilsan Paik Hospital | Goyang, Korea, Republic of |
| Hanyang University Guri Hospital | Guri, Korea, Republic of |
| Bundang Medical Center, CHA University | Seongnam, Korea, Republic of |
| Uijeongbu St. Mary's Hospital | Uijeongbu, Korea, Republic of |
| Samsung Changwon Medical Center | Changwon, Korea, Republic of |
| Wonkwang University Hospital | Iksan, Korea, Republic of |
| Chonbuk National University Hospital | Jeonju, Korea, Republic of |
| Kangwon National University Hospital | Chuncheon, Korea, Republic of |
| Chang Won Fatima Hospital | Changwon, Korea, Republic of |
| Dongguk University International Hospital | Goyang, Korea, Republic of |
| Hallym University Sacred Heart Hospital | Anyang, Korea, Republic of |
| Seoul National University Bundang Hospital | Seongnam, Korea, Republic of |
| Ajou University Hospital | Suwon, Korea, Republic of |
| Manila Doctors Hospital | Manila, Philippines |
| University of Santo Tomas | Manila, Philippines |
| The Medical City | Pasig, Philippines |
| St. Luke's Medical Center | Quezon City, Philippines |
